# Physiologic requirement for iron in pregnant women, assessed using the stable isotope tracer technique

**DOI:** 10.1186/s12986-020-00452-0

**Published:** 2020-04-21

**Authors:** Jie Cai, Tongxiang Ren, Jiaxi Lu, Jinghuan Wu, Deqian Mao, Weidong Li, Yu Zhang, Min Li, Jianhua Piao, Lichen Yang, Yuxia Ma, Jun Wang, Xiaoguang Yang

**Affiliations:** 1 The Key Laboratory of Trace Element Nutrition, National Institute for Nutrition and Health, Chinese center for disease control and prevention, 29 Nan Wei Road, Xicheng District, Beijing, 100050 People’s Republic of China; 2 Hospital Management Institute, Zhongnan Hospital of Wuhan University, 169 Donghu Road, Wuchang District, Wuhan, Hubei 430071 People’s Republic of China; 3 National Institute of Metrology, National Research Center for Certified Reference Material, No.18, Bei San Huan Dong Lu, Chaoyang District, Beijing, 10050 People’s Republic of China; 4 Hebei Medical University, 361 Zhongshan East Road, Shijiazhuang, Hebei 050017 People’s Republic of China

**Keywords:** Iron, Physiologic requirement, Pregnant women, Stable isotope tracer technique

## Abstract

**Background:**

Knowledge of the physiologic requirement for iron, the core index for the formulation of a dietary reference intake (DRI), is of great importance for the health of a pregnant woman and her fetus, and can help a mother accurately plan her iron supplementation. However, direct measurements of the physiologic requirement for iron during pregnancy are still lacking.

**Method:**

Eleven women of reproductive age from Hebei Province, China, who planned to become pregnant in the near future, were enrolled between January and March 2015 and included in the final analysis. Subjects participated in a 2-week metabolic trial in which they consumed 50 mg of the stable isotope ^58^Fe, and were then followed for ~ 2 years. The abundance of ^58^Fe and the total iron concentration in the circulation were measured using Multi-collector Inductively-Coupled Plasma Mass Spectrometry and Atomic Absorption Spectroscopy, respectively. The physiologic requirement for iron during pregnancy was then calculated by the formula derived from our previously published work.

**Results:**

The mean physiologic requirement for iron in the 11 subjects, across their entire pregnancies, was 3.05 mg.d^− 1^ in total and 44.0 μg.kg^− 1^.d^− 1^ after adjustment for body mass. The physiologic requirements for iron in the first, second, and third trimesters were 2.04 mg.d^− 1^, 3.26 mg.d^− 1^, and 4.13 mg.d^− 1^, respectively. When adjusted for body mass, the physiologic requirements for iron in different trimesters were 32.3 μg.kg^− 1^.d^− 1^, 46.9 μg.kg^− 1^.d^− 1^, and 55.7 μg.kg^− 1^.d^− 1^, respectively.

**Conclusion:**

We preliminarily explored the physiologic requirement for iron in pregnant women. The data demonstrated that pregnant women needed about twice iron than non-pregnant women. This research may be helpful for the design of future studies and the modification of iron DRIs.

**Trial registration:**

ChiCTR, ChiCTR-OCH-14004302. Registered 14 February 2014, http://www.chictr.org.cn/showproj.aspx?proj=5267

## Introduction

The nutritional status of pregnant women with respect to iron is of great importance for both maternal health and fetal nutrition, and inadequate supply of iron leads to iron deficiency anemia (IDA). According to the World Health Organization report on anemia [1], 38.2% of pregnant women worldwide develop anemia, predominantly as a result of iron deficiency (ID), and the Chinese national nutrition survey has shown that the prevalence of anemia in pregnant women in China was 17.2% in 2010–2012 [2], indicating that the iron status of pregnant women around the world is sub-optimal.

During pregnancy, the physiologic demand for iron increases markedly because of the expansion of red blood cell mass, and the requirement to secure an adequate iron supply for placental function and the growing fetus [3]. The iron store of pregnant women is often insufficient to meet these great requirements [4], and if combined with inadequate iron absorption, this gives rise to ID, and potentially IDA. IDA can lead to a series of negative health outcomes, such as low birth mass [5] and higher mortality of parturient mothers and newborns [6, 7]. In recent years, iron supplementation has been widely recommended for pregnant women to prevent IDA [8], but the level of supplementation is important, because excessive iron intake is also associated with health problems, such as cardiovascular disease, pancreatic damage, neurological disease and cancers [9–11]. The appropriate level of iron supplementation is dependent on the use of accurate dietary reference intakes (DRIs), a set of recommended values calculated based on measured physiologic requirements and absorption rates. However, due to the lack of direct experimental data regarding the physiologic requirement for iron in pregnant women, the iron DRIs that are currently in use are derived from values calculated for non-pregnant women, which based on the direct experimental data for men [12].

After absorbed by body, most of iron combines with the globin and then exists in red blood cells to participate in oxygen transport, while a small part of iron exists in myoglobin to participate in tissue respiration or be stored for emergency needs [13, 14]. There is no controlled mechanism in human body for the excretion of iron and the iron balance mainly relies on the regulation by iron absorption [13, 15]. Most iron is excreted by the digestive tract and a very small amount of iron is excreted in urine and sweat [16]. The menstruation and pregnancy bleeding of women will also excrete iron. The physiologic requirement for iron in adult men with a stable body mass is usually regarded as being equal to iron loss [12].

Measurements of the physiologic requirement for iron have already been carried out in toddlers, young men, and non-pregnant women [16–19], but not in pregnant women. These studies all have used an iron isotope, whether radioactive or stable, to trace the daily loss of iron and extrapolate the physiologic requirement for the study participants [16–19]. With technologic progress and improvements in ethical standards, the use of the stable isotopes, ^57^Fe and ^58^Fe, has completely replaced the use of radioisotopes in recent years [18, 19]. We have used ^58^Fe to establish an appropriate means of calculating the iron physiologic requirement in young adults and women of child-bearing age [18, 20]. In this study, we aimed to use this approach to assess the physiologic requirements for pregnant women for the first time.

## Materials and methods

### Subjects and experimental design

Eighty-two non-pregnant women of reproductive age lived in Xingtang County, Hebei Province, were planned to be enrolled in this study between January and March 2015. Participants were included if they were aged 20–35 years and planned to become pregnant in the near future. Potential participants were excluded if they: 1. had a disease that could affect iron absorption or metabolism (such as malabsorption, gastrointestinal ulcer, or inflammatory disease), or had abnormal iron nutritional status; 2. regularly took medication that could affect iron absorption or metabolism; 3. they were already pregnant before the study or menstruating during the metabolic trial.

**Fig. 1:**
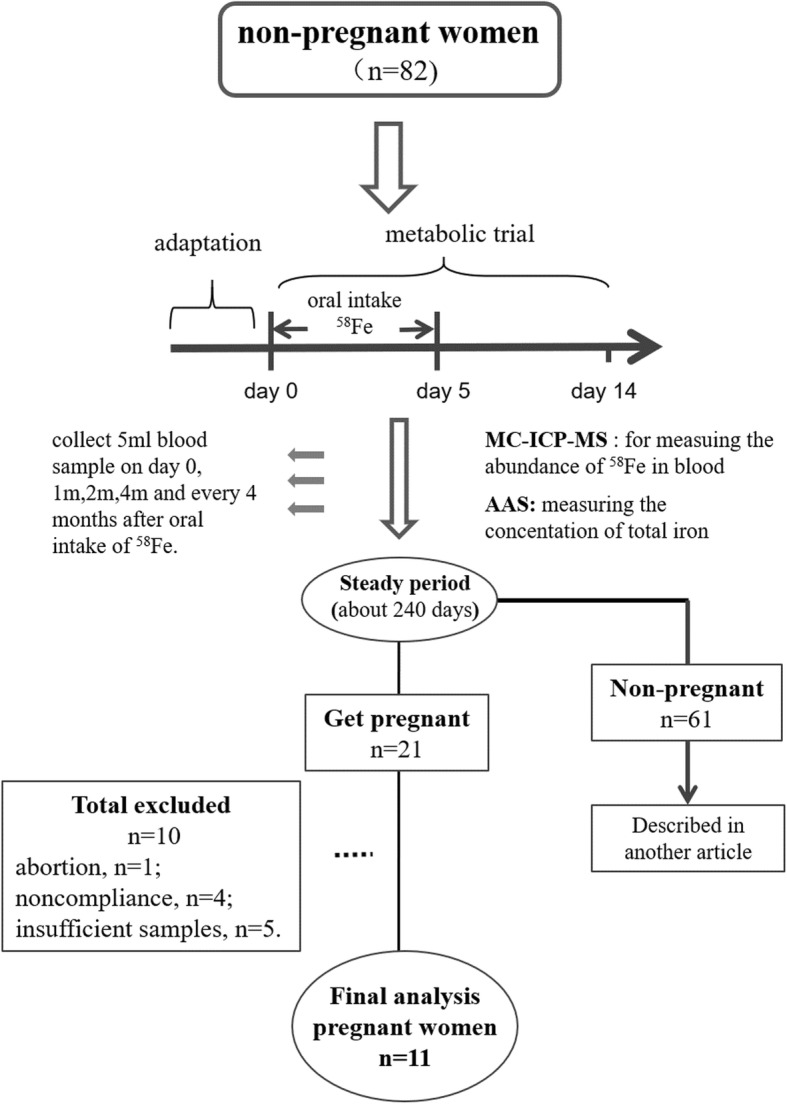
Flow chart

After 2 days of adaptation, a 2-week metabolic trial began during which they consumed ^58^Fe in their meals, in order to trace their iron metabolism. It was anticipated that the women would become pregnant after the absorbed iron had equilibrated within their bodies. Participants who then gave birth were followed for 3 months after delivery. The participants who did not become pregnant during the study were also followed for about 2 years and the results of them were published in the previous article [20]. The flow chart of this study was shown in Fig. 1.

The participants consumed ^58^Fe in meals in the form of ^58^FeSO_4_, 10 times over 5 days, providing a total of ~ 50 mg, at the beginning of the trial. During this 2-week period, the participants stayed in arranged accommodation to ensure compliance with the regimen. Diet and stool samples during the 2-week metabolic trial were collected every day to quantify the absorption of iron according to the metabolic balance method. Venous blood samples were collected to measure the ^58^Fe abundance and total iron concentration at the beginning and the ending of the metabolic trial. After the metabolic trial, participants would still be followed and provide venous blood samples at the first month, second month, forth month and every 4 months. Throughout the follow-up period, close attention was paid to the health of the participants, to ensure that they did not exhibit symptoms related to blood loss or abnormal iron metabolism. The trial was approved by the Ethics Committee of the National Institute of Nutrition and Health, Chinese Centers for Disease Control and Prevention, and registered at the Chinese Clinical Trial Registry (No: ChiCTR-OCH-14004302). Written informed consent was obtained from all subjects prior to their participation.

### Sample analysis

Blood samples were initially acid-digested using a Microwave Digestion System (Mars 6, GEM, USA) and 70% HNO_3_ solution. The digestion procedure was as follows: 120 °C: ramp 6 min, hold 5 min; 150 °C: ramp 5 min, hold 15 min; 190 °C: ramp 5 min, hold 30 min; 1600 W. Total iron concentration was quantified using atomic absorption spectroscopy (AAS) (PinAAcle 900, PerkinElmer). The abundance of ^58^Fe was analyzed using multi-collector inductively-coupled plasma mass spectrometry (MC-ICP-MS), and a standard-sample bracketing method. A mixture of argon and H_2_ was used as the collision gas to eliminate interference [21]. Under optimized conditions, the precision was 0.01–0.03% (relative standard deviation, RSD). Iron biochemical indices were measured using an automated iron biochemical analyzer (Hitach7180, Japan): serum ferritin (SF), unsaturated iron-binding capacity (UIBC), serum iron (SI), and transferrin (TRF). Inflammatory markers were also measured (C-reactive protein (CRP) and α- acid glycoprotein (α-AGP)).

### Calculations

The following formula was used to calculate the physiologic requirement for iron, which was based on the change in abundance of ^58^Fe during a set period of time, and the calculation of the mean iron requirement across various time periods. The formula was derived in our previously published work [18].

1 $$ \mathrm{R}=\mathrm{T}\times \mathrm{V}\times \left({\mathrm{P}}_{\mathrm{i}}-{\mathrm{P}}_{\mathrm{i}+\mathrm{t}}\right)\div \mathrm{t}\div \left[\left({\mathrm{P}}_{\mathrm{i}}+{\mathrm{P}}_{\mathrm{i}+\mathrm{t}}\right)/2-\mathrm{NA}\right]\div \mathrm{C} $$

where T is the total iron concentration in the blood (mg. L^− 1^), P_i_ is the isotopic abundance on day i, NA is the natural abundance of the isotope, t is the study period (days), R is the daily loss or intake of iron (mg), and V is the blood volume (L), which was calculated using the formula published by Carlsen and Bruun [22]:

V (ml) = (45.2 + 25.3 × exp.(− 0.0198 × DDW)) × BM (kg),

where DDW = 100 × (BM (kg) − 7.582 × exp.(0.01309 × BH (cm))) / 7.090(0.01309 × BH (cm)).

C is the iron circulation rate (the proportion of iron in the blood compared with that in the entire body), which was previously calculated to be 80.4% for non-pregnant women. Further details are given in Table S1.

We were unable to measure the body mass of every individual in each trimester of pregnancy. Therefore, we estimated the body mass in specific trimester of pregnancy using the measured baseline body mass and the expected increase in mass in each trimester of pregnancy according to the American Medical Association data [23]. The expected increase in mass varies with BMI, such that the mean expected weekly mass gains are 0.22 kg, 0.28 kg, 0.425 kg, and 0.51 kg when the BMI is > 30 kg.(m^2^)^− 1^, 25.0–29.9 kg.(m^2^)^− 1^, 18.5–24.9 kg.(m^2^)^− 1^, and < 18.5 kg.(m^2^)^− 1^, respectively (Table S2).

### Statistical analysis

Statistical analysis was performed using SAS 9.3 (SAS Institute Inc., Cary, NC, USA). The normality of the data was assessed using the Kolmogorov-Smirnoff test. Variables that conform to a normal distribution are expressed as mean ± SD and others are expressed as median (lower quartile, upper quartile). Differences between two groups were evaluated using Student’s *t*-test and among more than two groups using Analysis of Variance (ANOVA). The rank sum test was used to compare CRP and TFR values on different days. *P* < 0.05 was considered to represent statistical significance.

## Results

### Baseline information and follow-up

**Fig. 2:**
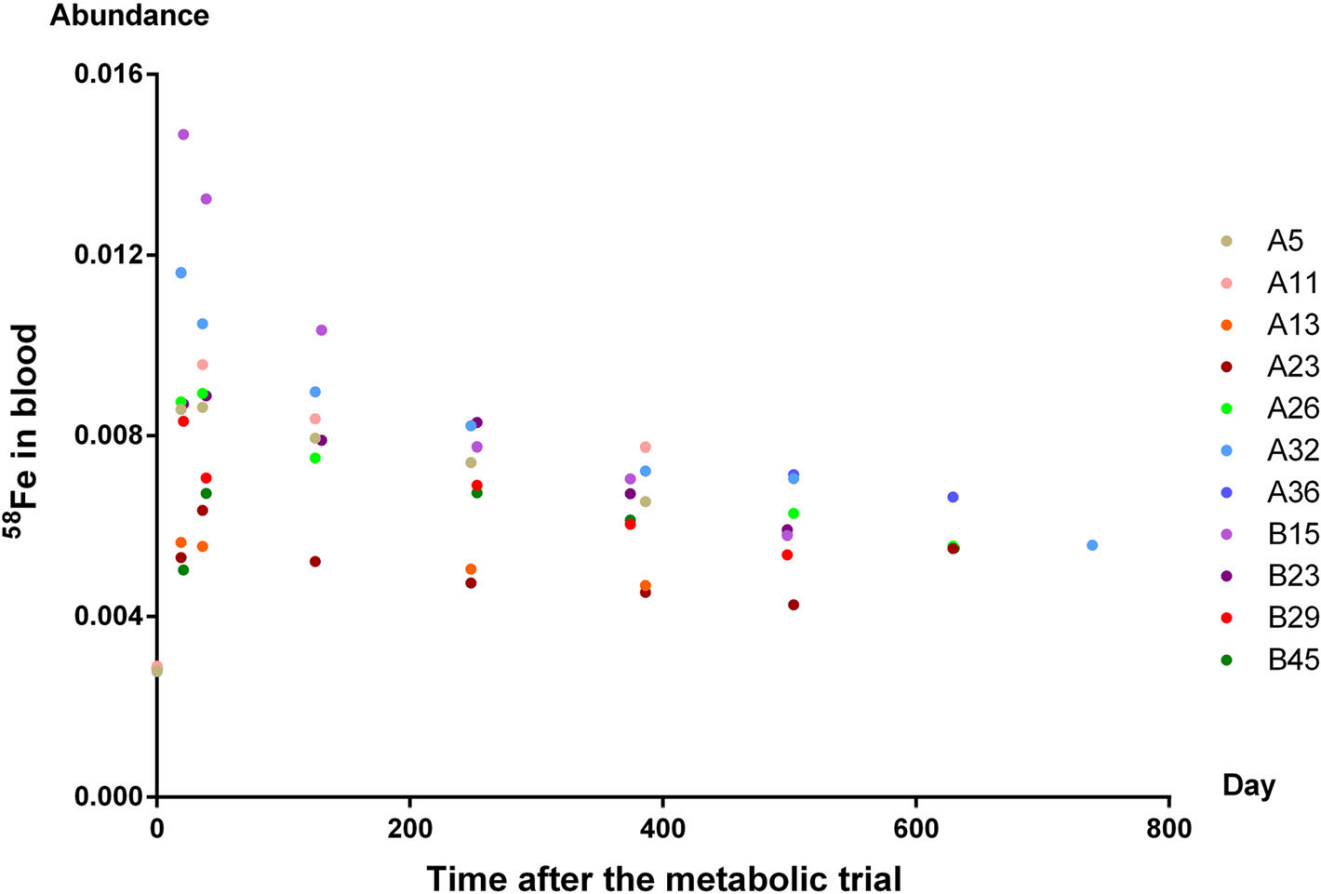
The abundance of ^58^Fe of pregnant women

At the beginning of the study, 82 non-pregnant women were enrolled, and 21 became pregnant during follow-up. Of these 21, one underwent a spontaneous abortion in her first trimester, no blood samples were obtained from four, and only one blood sample was obtained from five participants, which was insufficient to contribute to the iron requirement calculation. Therefore, 11 participants remained for whom data derived from two or more blood samples could be included in the analysis. The abundance of ^58^Fe was measured in blood samples from these 11 pregnant women. As shown in Fig. 2, the abundance of ^58^Fe increased rapidly after consumption of the isotope began, peaked at around 20 days, and remained stable for after 240 days.

The mean height was 157.5 cm at baseline, the mean body mass was 62.5 kg, the mean BMI was 24.8 kg.(m^2^)^− 1^, the mean age was 27 years, and the median time from the commencement of the study to getting pregnant was 335 days. Additional information is shown in Table 1.

**Table 1:** Baseline Information of Subjects

ID	Height (cm)	Weight (kg)	BMI (kg.(m^2^)^− 1^)	Age (y)	Pregnancy from when trial started (day)
A5	160.7	84.55	32.74	31	158
A11	166.0	52.40	19.00	24	120
A13	167.7	87.15	30.90	29	91
A23	158.2	53.50	21.30	27	335
A26	159.7	55.65	21.80	31	422
A32	154.8	59.90	25.00	22	336
A36	151.2	64.18	25.06	30	419
B15	152.3	61.03	26.31	25	360
B23	159.3	67.19	26.48	29	139
B29	150.6	46.35	20.44	26	75
B45	152.0	55.03	23.82	24	458
Average	157.5 ± 5.6	62.45 ± 12.34	24.80 ± 4.05	27 ± 3	335 (130, 390)

### Monitoring of the iron nutritional status of the participants

We measured iron biochemical indicators in these 11 pregnant women to evaluate their iron nutritional status in each trimester of pregnancy. The test results of 20 serum samples (more than one sample per person) showed that most of them were within the normal range (SF:12 ~ 150 μg. L^− 1^, UIBC:31.0 ~ 51.0 μmol. L^− 1^, SI:9.0–27.0 μmol. L^− 1^, TRF:1.93〜3.98 g.L^− 1^, CRP:< 5 mg. L^− 1^, α-AGP:0.55 ~ 1.4 g.L^− 1^) in each trimester (Table 2).

**Table 2:** Iron Nutritional Status of Pregnant Women

Trimester	N	SF (μg. L^−1^)	UIBC (μmol. L^− 1^)	SI (μmol. L^− 1^)	TRF (g.L^− 1^)	CRP (mg. L^− 1^)	α-AGP (g.L^− 1^)
First	7	12.57 ± 6.73	25.71 ± 10.78	10.51 ± 6.31	1.9(1.04,2.05)	0.3(0.2,0.8)	0.6 ± 0.2
Second	7	12.71 ± 5.41	43.71 ± 19.15	9.11 ± 2.84	2.39 (2.05,2.67)	3.6 (1.8,5.7)	0.6 ± 0.2
Third	6	20.8 ± 31.43	31.67 ± 19.11	12.7 ± 13.87	2.32 (1.88,2.55)	4.1 (1.8,5.1)	0.6 ± 0.6

### Physiologic requirement for iron in pregnant women

Blood samples from two adjacent time points were selected to calculate the physiologic requirement for iron. The mean physiologic requirement across the entire pregnancy of the 11 participants was 3.05 mg.d^− 1^ and the mean requirement adjusted for body mass was 44.0 μg.kg^− 1^.d^− 1^. The detailed findings are shown in Table 3.

**Table 3:** Physiologic Requirements for Iron in 11 Pregnant Women

ID	Trimester	Baseline weight (kg)	Baseline BMI (kg.(m^2^)^−1^)	Estimated weight in calculation period (kg)	Iron physiological requirement (μg.d^−1^)	Iron physiological requirements adjusted by weight (μg.kg^− 1^.d^− 1^)
A5	Second	84.55	32.74	89.55	3714.41	41.48
A11	First	52.40	19.00	60.69	1143.55	18.84
A13	Third	87.15	30.90	94.25	3842.51	40.77
A23	Second	53.50	21.30	60.15	3556.91	59.14
A26	Second	55.65	21.80	64.39	3752.52	58.28
A32	First	59.90	25.00	64.24	820.45	12.77
A36	Second	64.18	25.06	70.06	2011.76	28.72
B15	First	61.03	26.31	64.11	4813.28	75.08
B23	Third	67.19	26.48	74.21	4802.89	64.72
B29	Third	46.35	20.44	60.89	3758.01	61.72
B45	First	55.03	23.82	61.46	1379.00	22.44
$\overline{\mathrm{X}}$		62.45	24.81	69.45	3054.12	43.99
SD		12.94	4.25	11.93	1448.40	21.13

**Fig. 3:**
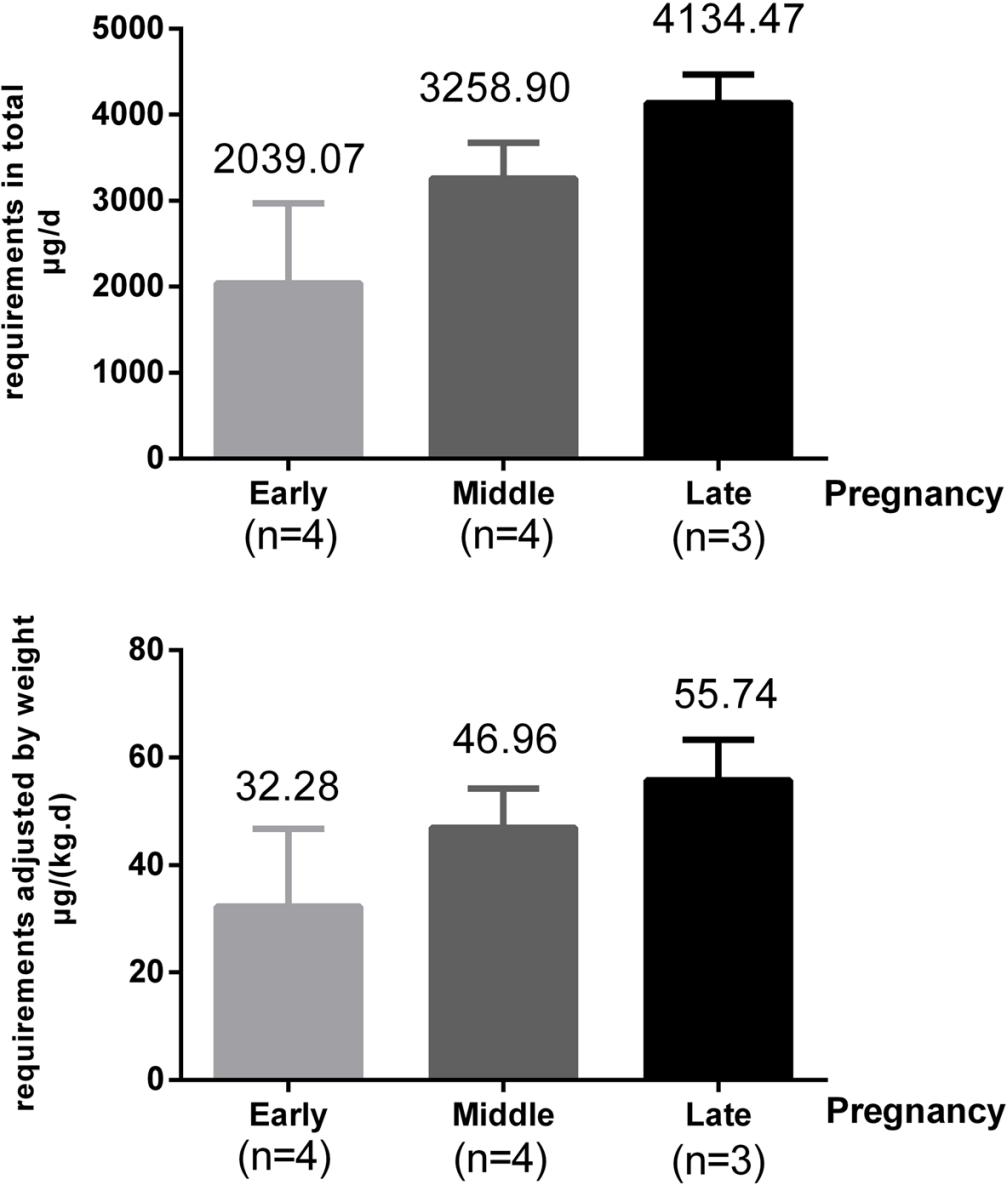
The physiologic requirements for iron of pregnant women

The physiologic requirements for iron in the first, second, and third trimesters were 2.04 mg.d^− 1^, 3.26 mg.d^− 1^, and 4.13 mg.d^− 1^, respectively. When adjusted for body mass, the requirements were 32.3 μg.kg^− 1^.d^− 1^, 46.9 μg.kg^− 1^.d^− 1^, and 55.7 μg.kg^− 1^.d^− 1^, respectively. Both the raw and adjusted iron requirements changed with the progression of pregnancy were shown in Table 4 and Fig. 3.

**Table 4:** Physiologic Requirements for Iron in Different Pregnancy Periods

Trimester	N	Iron physiological requirement (μg.d^−1^)	Iron physiological requirements adjusted by weight (μg.kg^−1^.d^−1^)
First	4	2039.07 ± 1863.59	32.28 ± 28.81
Second	4	3258.90 ± 835.73	46.90 ± 14.6
Third	3	4134.47 ± 580.41	55.74 ± 13.05

## Discussion

The iron nutritional status of the pregnant women in the present study was in the normal range throughout their pregnancies. The physiologic changes that occur during pregnancy include changes in the requirement for and metabolism of iron. In addition to a requirement for iron to support the greater blood volume, pregnant women also require additional iron to supply the fetus and placenta, and to create a reserve to replace that lost during childbirth. Previous studies have shown that SF and SI concentrations gradually decrease during pregnancy [24, 25], while serum TFR can slightly increase by the third trimester in iron-replete individuals [26]. Most of the iron transfer to the fetus occurs during the third trimester [27], which coincides with the lowest maternal hepcidin expression, permitting the maximum rate of iron supply to the maternal circulation, also facilitated by the steady increase in maternal TFR production during pregnancy [26]. The findings for the 11 pregnant women in our study showed no consistent trend, mainly because the data obtained during each trimester were not from the same individuals, and therefore could not reflect accurate longitudinal changes. However, the mean values of the iron biochemical indicators were still capable of demonstrating that the iron nutritional status was normal in the study cohort.

The total physiologic requirements of pregnant women over 18 years old for iron stated in DRIs by the Chinese Society of Nutrition are 1.09 mg.d^− 1^ in the first trimester, 4.62 mg.d^− 1^ in the second trimester, and 5.50 mg.d^− 1^ in the third trimester [28]. The requirements calculated in our study demonstrate a consistent trend towards an increase with the progress of pregnancy. However, we have shown a higher requirement in the first trimester and a lower requirement in the second and third trimesters than the Chinese DRIs. Due to the small sample size of each trimester, this part of the content could be further verified in the future researches. At present, the DRIs for the iron requirement of pregnant women from the USA and Canada [12], Australia and New Zealand [29], Japan [30], and Southeast Asia [31] have all been estimated using the basal loss of iron in men or non-pregnant women, rather than using data derived from direct measurements conducted in pregnant women.

Although no previous studies have performed measurements in pregnant women, the requirements for toddlers, adult men, and non-pregnant women have been reported [16–20, 32, 33]. These showed the mean requirements to be ~ 1 mg.d^− 1^ and 14 μg.kg^− 1^.d^− 1^ for adult men, and 1.2 ~ 1.5 mg.d^− 1^ and 21 ~ 24 μg.kg^− 1^.d^− 1^ for women at reproductive age. The present study has shown that the mean requirement for pregnant women across their entire pregnancies is 3.05 mg.d^− 1^, and 44.0 μg.kg^− 1^.d^− 1^ after adjustment for body mass, which is about two times the iron requirement for women at reproductive age. The greater need for iron in pregnant women can mainly be ascribed to the expansion of maternal blood volume, the demands of tissue synthesis in the fetus, and the creation of an iron reserve during pregnancy. Meanwhile, pregnant women do not lose iron as a result of menstruation, which offsets the additional requirement for iron to a certain extent.

The present study assessed the physiologic requirement for iron of pregnant women for the first time. However, it also had some limitations. Firstly, pregnant women demonstrate modifications to their usual iron metabolism, but there is few evidence to suggest that they are in iron homeostasis and that their mean daily iron loss is similar to their iron requirement. Because of the lack of existing methods to evaluate the physiologic requirement for iron in pregnant women, we adopted the method used for studying adult men and non-pregnant women, which should be further validated in the future. Secondly, we estimated the body mass of the participants in their three trimesters because we were unable to obtain their actual body masses during the follow-up period, which might negatively affect the accuracy of the data. Thirdly, because it was difficult to collect blood during pregnancy, we could not calculate the requirements in each trimester for the same individual, in order to make longitudinal comparisons. Thus, the data for the three trimesters reflect a general trend, rather than a precise dynamic change. Furthermore, the sample size of each trimester is relatively small, so we have not made the statistical comparison but just showed the figures. Therefore, the changes of iron demand in different pregnancy periods could be further verified in future researches.

It should be noted that, the special physiological conditions of pregnant women brought difficulties to the trial and follow-up, resulting in the relatively small sample size of this study. Even so, our results are comparable with the same data provided by other studies [32, 33]. In fact, the sample size of this type of study is limited by the acceptability of isotopes and the long-term follow-up; for example, the study by Finch in 1959 [32] only reported the daily iron loss of 12 nonmenstruating women and 6 menstruating women, and in the study by Janet R Hunt in 2009 [33], there was only 15 menstruating women and 5 postmenopausal women included. The data on pregnant women is even lacking. Despite some limitations, our findings still represent a significant step forward, both with regard to the methodology and the data generated. The calculated physiologic requirements for iron in pregnant women might be helpful for the design of future studies and the modification of iron DRIs.

## Conclusion

In this study, the physiologic requirements for iron in pregnant women were found to be 3 mg.d^− 1^ and 44 μg.kg^− 1^.d^− 1^ across the entire pregnancy, which are about twice of that in non-pregnant women.

## Supplementary information

**Additional file 1: Table S1.** The Circulation Rate of Non-pregnant Women.

**Additional file 2: Table S2.** Estimation of Maternal Weight Gain during Pregnancy.

## Data Availability

The datasets used and/or analyzed during the current study are available from the corresponding author on reasonable request.

## Abbreviations

AAS: Atomic Absorption Spectroscopy
ANOVA: Analysis of Variance
α-AGP: α- acid Glycoprotein
CRP: C-reactive Protein
DRI: Dietary Reference Intake
ID: Iron Deficiency
IDA: Iron Deficiency Anemia
MC-ICP-MS: Multi-collector Inductively-coupled Plasma Mass Spectrometry
RSD: Relative Standard Deviation
SF: Serum Ferritin
SI: Serum Iron
TRF: Transferrin
UIBC: Unsaturated Iron-binding Capacity
