# Supplementary material for: Physiologic requirement for iron in pregnant women, assessed using the stable isotope tracer technique
Source: Nutr Metab (Lond). 2020 Apr 21;17:33. doi: 10.1186/s12986-020-00452-0 (PMC7175517; doi:10.1186/s12986-020-00452-0)
Supplement: Supplementary file 1 — Additional file 1: Table S1. The Circulation Rate of Non-pregnant Women. [file 12986_2020_452_MOESM1_ESM.docx]

| Table S1. The Circulation Rate of Non-pregnant Women | | | | | |
| --- | --- | --- | --- | --- | --- |
| Group | N | Age | Weight (kg) | BMI (kg/m^2^) | Circulation rate (%) |
| 1 | 15 | 29.27±4.33 | 68.65±11.41 | 27.02±4.16 | 79.44±4.41 |
| 2 | 31 | 30.07±4.19 | 59.72±8.28 | 23.38±3.10 | 80.86±4.29 |
| Total | 46 | 29.79±4.21 | 62.63±10.2 | 24.57±3.84 | 80.4±4.33 |
| t |  | -0.591 | 3.023 | 3.328 | -1.044 |
| *P* |  | 0.558 | 0.004 | 0.002 | 0.302 |
