# Supplementary material for: Physiologic requirement for iron in pregnant women, assessed using the stable isotope tracer technique
Source: Nutr Metab (Lond). 2020 Apr 21;17:33. doi: 10.1186/s12986-020-00452-0 (PMC7175517; doi:10.1186/s12986-020-00452-0)
Supplement: Supplementary file 2 — Additional file 2: Table S2. Estimation of Maternal Weight Gain during Pregnancy. [file 12986_2020_452_MOESM2_ESM.docx]

| Table S2. Estimation of Maternal Weight Gain during Pregnancy | | |
| --- | --- | --- |
| BMI (kg/m^2^) | Weekly weight gain (kg) | Average weekly gain (kg) |
| ≧30 | 0.17-0.27 | 0.22 |
| 25.0~29.9 | 0.23-0.33 | 0.28 |
| 18.5~24.9 | 0.35-0.50 | 0.425 |
| <18.5 | 0.44-0.58 | 0.51 |
